# Complete Genome Sequences of Two Salmonella Viruses, VSe11 and VSe102 (Family *Myoviridae*, Subfamily *Ounavirinae*), with a Very High Degree of Similarity

**DOI:** 10.1128/genomeA.00398-18

**Published:** 2018-05-24

**Authors:** Nikolay V. Volozhantsev, Egor A. Denisenko, Angelina A. Kislichkina, Vera P. Myakinina, Valentina M. Krasilnikova, Vladimir V. Verevkin, Lidiya A. Kadnikova, Nadezhda V. Maiskaya, Alexandr G. Bogun, Ivan A. Dyatlov

**Affiliations:** a State Research Center for Applied Microbiology and Biotechnology, Obolensk, Moscow Region, Russia

## Abstract

Two lytic double-stranded DNA bacteriophages, VSe11 and VSe102, infecting broad-spectrum Salmonella enterica were isolated from the sewage of two different poultry farms. The phage genomes comprise 86,360 bp and 86,365 bp, respectively, with a G+C content of 39.0%, and both contain 129 putative coding sequences.

## GENOME ANNOUNCEMENT

Nontyphoid Salmonella enterica is the most common cause of foodborne illnesses in humans, which remain an important health problem worldwide (1–3). The situation is compounded by the fact that Salmonella spp. commonly acquired from contaminated food have become resistant to various antibiotics and can pass on their resistance genes to other bacteria (4–6). The rise of antibiotic resistance is making it harder to treat Salmonella infections. Bacterial viruses are emerging as potential biocontrol agents against this pathogen (7–9).

Here, we report the genome sequences of the Salmonella bacteriophages VSe11 and VSe102, isolated from sewage samples collected in two different poultry farms in Moscow Region, Russia. VSe11 and VSe102 have a similar broad-spectrum lytic activity against Salmonella spp. of serovars Enteritidis, Typhimurium, and Infantis. At the same time, the phages have a minor difference in strain specificity and different efficiency of plating on some Salmonella strains.

The phage DNA were sequenced using Ion Torrent PGM (Life Technologies, Inc., USA). A total of 16,861 (for VSe11) and 21,319 (for VSe102) reads were generated and assembled into single contigs using Newbler 2.9.

The circularly permuted linear double-stranded DNA genomes of VSe11 and VSe102 phages have lengths of 86,360 bp and 86,365 bp, respectively, with a G+C content of 39.0% in both cases. Coding sequences (CDSs) within the phage genomes were allocated using the software tool GeneMarkS (10), and annotation was performed via the Rapid Annotations using Subsystems Technology server (11) and NCBI BLAST algorithms (12). It was shown that both phage genomes have 129 CDSs on both DNA strands and 21 genes encoding tRNAs for 14 amino acids.

Whole-genome-based analysis revealed that the VSe11 and VSe102 genomes have very high similarity. In total, 99.5% of their genome sequences are identical. Out of 129 CDSs, 125 potential genes encode proteins with identical amino acid sequences. Only four CDSs (087, 089, 090, and 091) have a nucleotide identity of 67% to 94% and encode hypothetical proteins that somewhat differ in the amino acid sequence. Perhaps this divergence determines a minor difference in strain specificity of the phages. One more difference between the VSe11 and VSe102 genomes is revealed in the noncoding region (between CDSs 021 and 022) containing repeat sequences. An additional repeat element of 33 bp was detected in this region of the phage VSe102 genome.

The BLASTn results showed that the closest neighbors of phages VSe11 and VSe102 are the Salmonella phages Mushroom (GenBank accession no. KP143762) (13), SPT-1 (JX181822), and Si3 (KY626162). All of them encode core proteins inherent to Felix O1-like phages of the family *Myoviridae* subfamily *Ounavirinae* (14). The VSe11 and VSe102 genomes, as well as those of Mushroom, SPT-1, and Vsi3, contain two genes encoding DNA polymerases. However, unlike the Mushroom and Si3 phages, VSe11 and VSe102 have an additional CDS corresponding to a recombination endonuclease VII in the region separating the two polymerase genes, identical to that of phage SPT-1.

### Accession number(s).

The VSe11 and VSe102 genome sequences are available in GenBank under the accession no. MG251391 and MG251392, respectively.
